# Pharmacological targeting of secondary brain damage following ischemic or hemorrhagic stroke, traumatic brain injury, and bacterial meningitis - a systematic review and meta-analysis

**DOI:** 10.1186/s12883-017-0994-z

**Published:** 2017-12-07

**Authors:** Thomas Beez, Hans-Jakob Steiger, Nima Etminan

**Affiliations:** 1 Department of Neurosurgery, Medical Faculty, Heinrich-Heine-University, Düsseldorf, Germany; 2 Department of Neurosurgery, Medical Faculty, University Hospital Mannheim, University of Heidelberg, Mannheim, Germany

**Keywords:** Ischemic stroke, Hemorrhagic stroke, Meningitis, Traumatic brain injury, Secondary brain damage, Neuroprotection, Randomized controlled trials

## Abstract

**Background:**

The effectiveness of pharmacological strategies exclusively targeting secondary brain damage (SBD) following ischemic stroke, aneurysmal subarachnoid hemorrhage, aSAH, intracerebral hemorrhage (ICH), traumatic brain injury (TBI) and bacterial meningitis is unclear. This meta-analysis studied the effect of SBD targeted treatment on clinical outcome across the pathological entities.

**Methods:**

Randomized, controlled, double-blinded trials on aforementioned entities with ‘death’ as endpoint were identified. Effect sizes were analyzed and expressed as pooled risk ratio (RR) estimates with 95% confidence intervals (CI). 123 studies fulfilled the criteria, with data on 66,561 patients.

**Results:**

In the pooled analysis, there was a minor reduction of mortality for aSAH [RR 0.93 (95% CI:0.85–1.02)], ICH [RR 0.92 (95% CI:0.82–1.03)] and bacterial meningitis [RR 0.86 (95% CI:0.68–1.09)]. No reduction of mortality was found for ischemic stroke [RR 1.05 (95% CI:1.00–1.11)] and TBI [RR 1.03 (95% CI:0.93–1.15)]. Additional analysis of “poor outcome” as endpoint gave similar results. Subgroup analysis with respect to effector mechanisms showed a tendency towards a reduced mortality for the effector mechanism category “oxidative metabolism/stress” for aSAH with a risk ratio of 0.86 [95% CI: 0.73–1.00]. Regarding specific medications, a statistically significant reduction of mortality and poor outcome was confirmed only for nimodipine for aSAH and dexamethasone for bacterial meningitis.

**Conclusions:**

Our results show that only a few selected SBD directed medications are likely to reduce the rate of death and poor outcome following aSAH, and bacterial meningitis, while no convincing evidence could be found for the usefulness of SBD directed medications in ischemic stroke, ICH and TBI. However, a subtle effect on good or excellent outcome might remain undetected. These results should lead to a new perspective of secondary reactions following cerebral injury. These processes should not be seen as suicide mechanisms that need to be fought. They should be rather seen as well orchestrated clean-up mechanisms, which may today be somewhat too active in a few very specific constellations, such as meningitis under antibiotic treatment and aSAH after surgical or endovascular exclusion of the aneurysm.

**Electronic supplementary material:**

The online version of this article (10.1186/s12883-017-0994-z) contains supplementary material, which is available to authorized users.

## Background

The term ‘secondary brain damage (SBD)’ refers to delayed detrimental functional and structural sequelae after various types of acute cerebral injury. The underlying mechanisms are triggered by the primary ictus, such as ischemic or hemorrhagic stroke (aneurysmal subarachnoid, aSAH or intracerebral hemorrhage, ICH) or traumatic brain injury (TBI) and intracranial infections [1–4]. Numerous mechanisms contributing to the pathogenesis of SBD following the aforementioned acute cerebral events have been identified over the past decades, which subsequently led to the development and clinical investigation of numerous experimental pharmacological treatments focused on different SBD mechanisms. SBD mechanisms include impairment of cerebral flood flow and cerebral autoregulation, metabolic dysfunction, edema formation, oxidative stress, disruption of the blood brain barrier and inflammation [1–4].

While some SBD pathomechanisms are specific for ischemic and hemorrhagic stroke, TBI and intracranial infections, other mechanisms are a rather unspecific reaction of the injured brain, such as edema and delayed ischemia. At the present time it remains unclear whether a pharmacological strategy targeting one specific pathomechanism could result in a beneficial effect on outcome [5, 6]. Moreover, the relevance of targeted treatment of SBD, in addition to established general measures of critical care, e.g. avoidance of arterial hypotension, increased intracranial pressure, hyperthermia, and hypoxemia, is uncertain [7–11]. We performed a systematic review and meta-analysis to summarize the currently available randomized clinical trials exclusively targeting specific pathomechanisms of SBD with the aim to improve clinical outcome following ischemic stroke, ICH, SAH, TBI, and bacterial meningitis. In order to obtain practically useful information we grouped the available phase III randomized clinical trials according to addressed pathomechanisms.

## Methods

For this systematic review we used the Cochrane Collaboration format [12] and followed the PRISMA checklist [13].

### Search strategy for identification of studies

The authors conducted a systematic search of the Pubmed database (http://www.ncbi.nlm.nih.gov/pubmed) in February 2015 for the terms ischemic stroke, brain infarction, subarachnoid hemorrhage, SAH, intracerebral hemorrhage, intracranial hemorrhage, ICH, traumatic brain injury, brain trauma, TBI and bacterial meningitis. The search was limited to ‘randomized controlled trials’, ‘human studies’ and ‘English’. Additionally, previous review articles or meta-analyses were searched for studies matching our inclusion criteria. The retrieved articles were screened for relevance in a step-wise manner: First the titles were reviewed, then the corresponding abstract and in case of further uncertainty the full-text was screened by the two first authors (TB and HJS) until all retrieved articles were either included or omitted.

### Types of studies

Randomized, blinded, controlled clinical trials studying the efficacy of pharmacological treatment to reduce poor outcome or death due to SBD following ischemic stroke, aneurysmal subarachnoid hemorrhage (aSAH), intracerebral hemorrhage (ICH), traumatic brain injury (TBI) and bacterial meningitis were included, irrespective of the type of treatment. All studies analyzing therapy of the primary pathomechanisms, e.g. thrombolysis and recanalization for ischemic stroke or antibiotics for meningitis were excluded. Studies potentially targeting both the primary pathomechanism and SBD, e.g. surgical evacuation of intracerebral hemorrhage, were excluded to avoid bias.

SBD was defined as all adverse events occurring during of the first three weeks following acute brain injury, i.e. all mechanisms that are triggered by the primary ictus, ultimately resulting in brain oedema, micro- and macrovascular narrowing or spasm, progressive cerebral infarction and/or hemorrhage [2, 4, 14]. On a molecular level such mechanisms comprise metabolic disturbance, inflammatory response, excitotoxicity, oxidative stress and apoptosis [1, 15, 16].

Trials with different outcome parameters other than death and/or poor neurological outcome as well as studies where outcome data could not be extracted due to inadequate reporting had to be excluded. Poor neurological outcome, i.e. severe disability and/or vegetative state or death was measured either using the modified Rankin Scale (mRS), the (extended) Glasgow Outcome Scale (GOS) or the Barthel Index (BI). If a study provided numbers on the individual categories, data were summarized according to the definition of poor outcome as a score of 3 to 6 on the mRS, 1 to 3 on the GOS or less than or equal to 70% on the BI. When an inverted GOS was used, data were readjusted to the original scale. If a study provided the percentage of patients with an outcome event, the actual numbers were calculated from the percentages.

### Grouping of included studies

The included trials were classified according to the addressed pathology; ischemic stroke, ICH, aSAH, TBI, and intracranial infections as well as according to the addressed pathomechanism or pharmacological class respectively. Here we distinguished the categories 1) blood pressure modification/vasodilatation, 2) hematology/immunology, 3) lipid metabolism, 4) neurotransmission, 5) oxidative metabolism/stress and 6) other.

### Statistics

Data were processed using the Review Manager 5.3.0 as supplied by the Cochrane Collaboration. Effect sizes were expressed as pooled risk ratio (RR) estimates. Analyses for death and poor outcome were performed for the entire type of injury cohorts as well as for subgroups according to common pharmacological classes. Statistical uncertainty was expressed in 95% confidence intervals (CI). Irrespective of the probability value of the I^2^ test, we exclusively used random-effects models for all pooled data analyses because of the heterogeneous sample sizes among the underlying patient populations.

Risk of bias of the included studies was assessed by reviewing methodology for allocation concealment and adequate blinding.

## Results

**Fig. 1:**
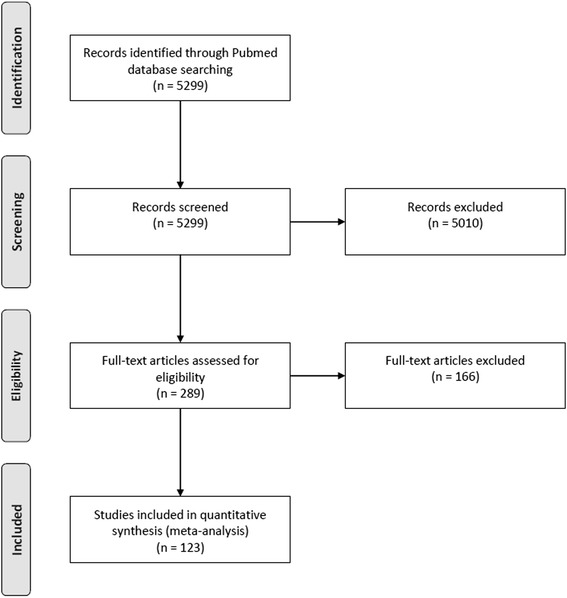
PRISMA flow chart for the current meta-analysis

Out of a total of 5299 reports, ultimately data from 123 studies targeting specific pathomechanisms of SBD fulfilled the specified criteria and the corresponding data on a total of 66,561 patients was included in the analysis (Fig. 1). The corresponding references for the included studies as well as the complete risk of bias assessment are provided in the online supplementary information (Additional file 1). Data on poor outcome was available in 67% of the included studies.

### Ischemic stroke meta-analysis

**Fig. 2:**
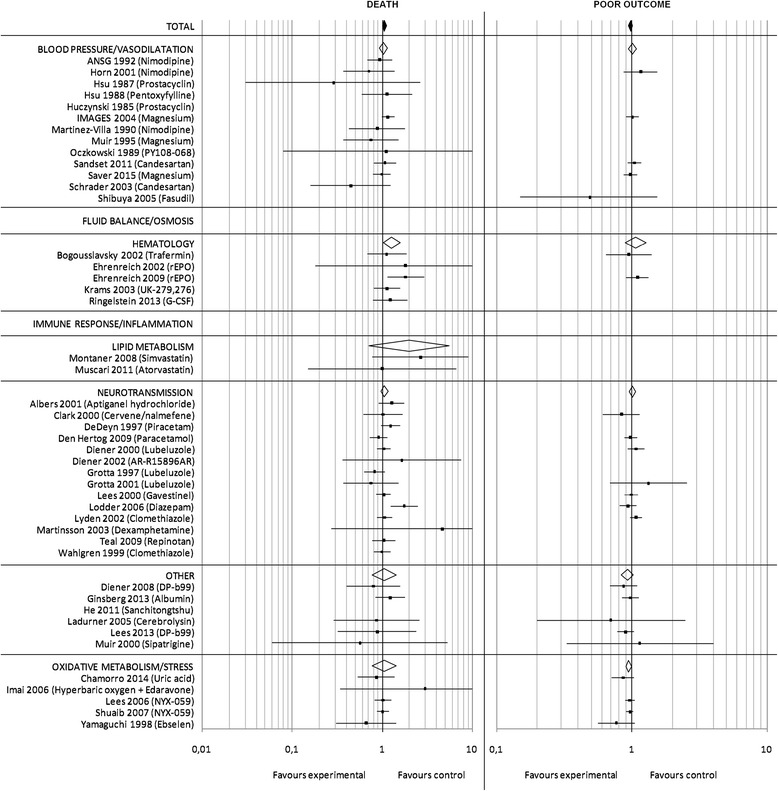
Ischemic stroke meta-analysis: Pooled RR and 95% CI estimates for death and poor outcome are illustrated for studies on ischemic stroke. The underlying treatment strategy is given in brackets. Studies are grouped according to common effector mechanisms and RR for subgroups are included. A detailed reference list is provided in Additional file 1

Out of a total of 2493 studies, ultimately 45 met our selection criteria. The majority of studies (*n* = 29) excluded patients with severe obtundation or coma. Several studies (*n* = 19) excluded patients with mild or no symptoms (i.e. NIHSS score < 4–8). Thus the typical patient enrolled in the majority of studies would have a moderate to severe ischemic stroke. A total of 30,435 patients were randomized into either experimental (15,904 patients) or control treatment (14,531 patients). The outcome was assessed after 3 months in 75.6% of studies. The overall risk ratio for death (Fig. 2) in the pooled analysis of all trials was 1.05 [95% CI: 1.00–1.11] (*p* = 0.05). For poor outcome (Fig. 2), data was available for 22,297 patients and the overall risk ratio in the pooled analysis was 0.98 [95% CI: 0.95–1.01] (*p* = 0.23). Two studies investigating the effect of erythropoietin and diazepam reported a significant detrimental effect with regard to death as endpoint [17, 18].

The subgroup analysis (Fig. 2) demonstrated a detrimental effect on death for the effector mechanism category “hematology/immunology” with a risk ratio of 1.27 [95% CI: 1.03–1.57] (*p* = 0.03).

### aSAH meta-analysis

**Fig. 3:**
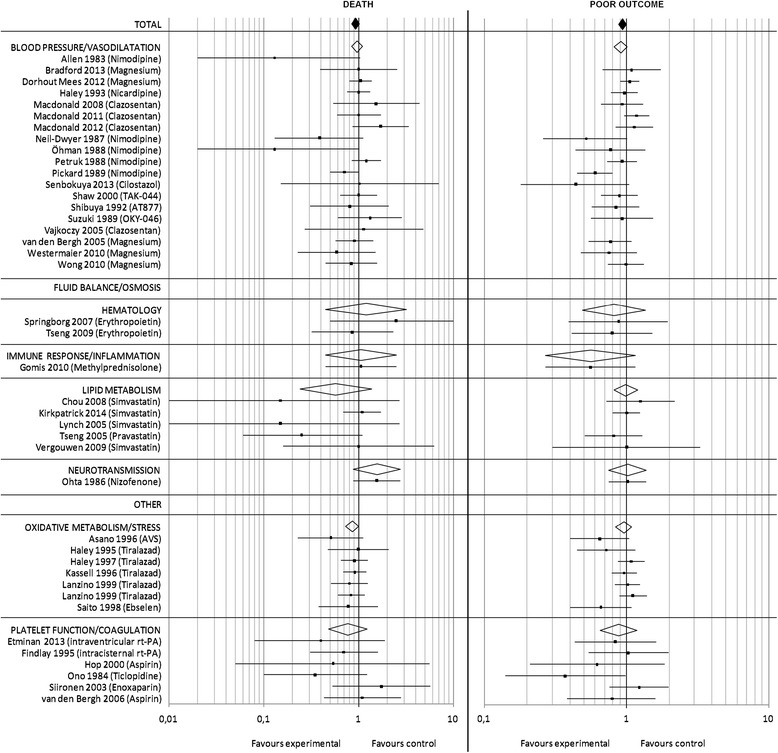
aSAH meta-analysis: Pooled RR and 95% CI estimates for death and poor outcome are illustrated for studies on aSAH. The underlying treatment strategy is given in brackets. Studies are grouped according to common effector mechanisms and RR for subgroups are included. A detailed reference list is provided in Additional file 1

For aSAH, the initial search yielded 370 reports, of which ultimately a total of 41 studies were included. The majority of studies (*n* = 24) enrolled all SAH patients irrespective of severity. Eleven studies excluded comatose or moribund patients (i.e. WFNS grade 5 or Hunt and Hess grade 5), four studies excluded good grade patients (i.e. WFNS grade 1). A total of 13,647 patients were randomized into experimental (7696 patients) or control treatment (5951 patients). The outcome was assessed after 3 months in 57.5% of studies. The overall RR for death (Fig. 3) in the pooled analysis of all trials was 0.93 [95% CI:0.85–1.02] (*p* = 0.13). For poor outcome (Fig. 3) data was available for 13,460 patients and the overall RR in the pooled analysis was 0.94 [95% CI: 0.88–1.00] (*p* = 0.05). There were no studies which demonstrated a significant beneficial or detrimental effect on the outcome measure ‘death’. Two studies reported a significant reduction of poor outcome [19, 20]. Regarding allocation concealment as well as adequate blinding the risk of bias was low in one and unclear in the other of these two studies. The risk of bias regarding allocation concealment as well as adequate blinding was unclear in this study.

The subgroup analysis (Fig. 3) showed a tendency towards a beneficial effect for the effector mechanism category “oxidative metabolism/stress” with RR 0.86 [95% CI: 0.73–1.00] (*p* = 0.05).

### ICH meta-analysis

**Fig. 4:**
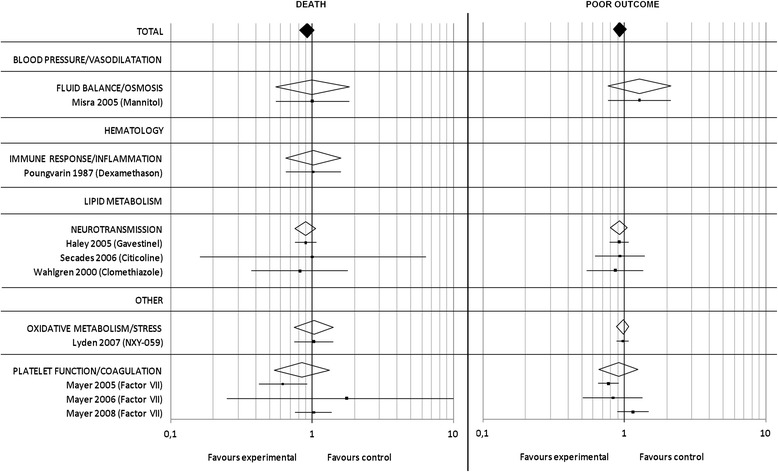
ICH meta-analysis: Pooled RR and 95% CI estimates for death and poor outcome are illustrated for studies on ICH. The underlying treatment strategy is given in brackets. Studies are grouped according to common effector mechanisms and RR for subgroups are included. A detailed reference list is provided in Additional file 1

Out of a total of 970 reports, 9 studies targeting specific SBD mechanisms after ICH were included. Four studies excluded comatose patients (i.e. GCS score < 5–8), three studies required patients to be awake and two studies enrolled all ICH patients. 2803 patients were randomized into either experimental (1665 patients) or control treatment (1138 patients). The outcome was assessed after 3 months in 88.9% of studies. The overall RR for death (Fig. 4) in the pooled analysis of all trials was 0.92 [95% CI 0.82–1.03] (*p* = 0.15). For poor outcome (Fig. 4) as an outcome measure, data was available for 2710 patients and the overall RR in the pooled analysis was 0.93 [95% CI: 0.84–1.03] (*p* = 0.19). One study demonstrated a significant reduction of mortality and poor outcome in patients receiving recombinant activated factor VII [21]. Regarding allocation concealment as well as adequate blinding the risk of bias was judged low in this study. However, the expected positive effect of recombinant activated factor VII could not be confirmed in the two subsequent studies.

The subgroup analysis (Fig. 4) showed no significant effects on death for any effector mechanism.

### TBI meta-analysis

**Fig. 5:**
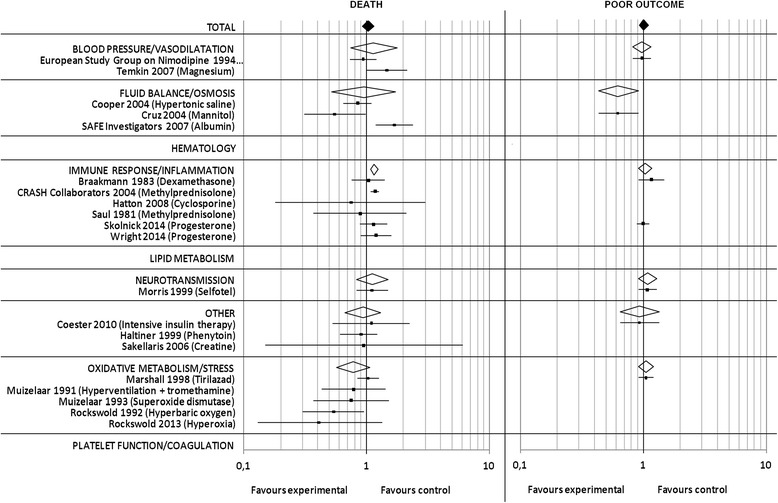
TBI meta-analysis: Pooled RR and 95% CI estimates for death and poor outcome are illustrated for studies on TBI. The underlying treatment strategy is given in brackets. Studies are grouped according to common effector mechanisms and RR for subgroups are included. A detailed reference list is provided in Additional file 1

A total of 20 out of 1052 studies on TBI met our inclusion criteria. The majority of studies (*n* = 18) enrolled patients with severe TBI, with five trials excluding patients presenting with fixed dilated pupils. Overall, 17,058 patients were randomized into experimental (8589 patients) or control treatment (8469 patients). The outcome was assessed after 6 months in 70.0% of studies. Overall, the RR for death (Fig. 5) was 1.03 [95% CI 0.93–1.15] (*p* = 0.58). For ‘poor outcome’ (Fig. 5) data was available for 4066 patients and the overall RR in the pooled analysis was 1.01 [95% CI: 0.93–1.10] (*p* = 0.85). One study investigating the effect of hyperbaric oxygen treatment and one trial of high dose mannitol treatment demonstrated a significant effect on reduction of mortality [22, 23]. Regarding the latter trial concerns have been raised regarding adherence to principles of good scientific practice [24]. Although the publication has not been formally retracted, caution should be exercised when interpreting these results. For allocation concealment the risk of bias was judged high in one, and unclear in the other study, and with regard to adequate blinding the risk of bias was considered high in both these studies. Two studies, one investigating the use of albumin for fluid resuscitation, the other methylprednisolone reported a significant detrimental effect on outcome [25, 26].

The subgroup analysis (Fig. 5) showed a detrimental effect for the effector mechanism category “immune response/inflammation”, with a RR of 1.16 [95% CI: 1.08–1.25] (*p* < 0.0001).

### Bacterial meningitis meta-analysis

Eight out of a total of 414 studies on bacterial meningitis, reporting on a total of 2618 patients, of which 1328 were randomized to experimental and 1290 to control treatment, met our inclusion criteria. All studies investigated the effect of corticosteroids in the context of bacterial meningitis.

**Fig. 6:**
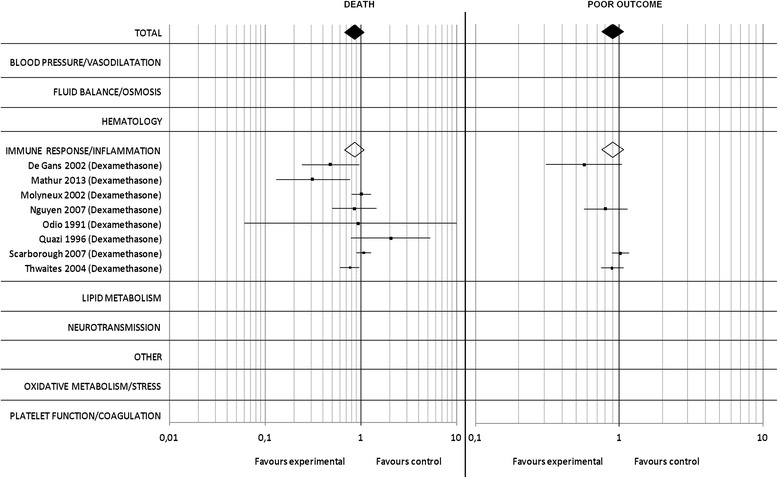
Bacterial meningitis meta-analysis: Pooled RR and 95% CI estimates for death and poor outcome are illustrated for studies on bacterial meningitis. The underlying treatment strategy is given in brackets. Studies are grouped according to common effector mechanisms and RR for subgroups are included. A detailed reference list is provided in Additional file 1

Overall, the RR for death (Fig. 6) in the pooled analysis was 0.86 [95% CI 0.68–1.09] (*p* = 0.22). For ‘poor outcome’ (Fig. 6) data was available for 1746 patients and the overall risk ratio in the pooled analysis was 0.90 [95% CI: 0.76–1.07] (p = 0.22). Three studies demonstrated a significant reduction of mortality [27–29].

For allocation concealment, risk of bias was low in 2 of these 3 studies, and high in 1 study. For adequate blinding, risk of bias was low in 2 studies, and high in 1 study.

## Discussion

While previous meta-analyses have investigated the effects of various experimental pharmacological or surgical treatments for specific cerebral diseases on different outcome measures [30–34], the present meta-analysis explores the efficacy of pharmacological treatments solely targeting specific SBD mechanisms across the various underlying entities. All entities analyzed in the present study are considered to share at least partially some common mechanisms of SBD [35–37]. In contrast to the initial ictus, e.g. aSAH, the mechanisms causing SBD are considered amenable for therapeutic intervention and have therefore been subject of considerable scientific efforts.

Our pooled analyses for the main pathological groups provided risk ratios of poor outcome or death for the experimental groups in a relatively narrow range from 0.86 to 1.03. For the subgroups of addressed pathways the risk ratios scattered somewhat wider with a range from 0.57 to 2.0. Since in such a pooled analysis the effect of positive concepts and result is diluted by a larger number of negative attempts, the relatively narrow range of the pooled results is not surprising.

For ischemic stroke, TBI and ICH the pooled risk ratios for the experimental groups were close to one, which leads to the conclusion that addressing SBD could not be confirmed as a relevant therapeutic target. There were only 3 outliers in these groups, trials showing a statistically significant benefit for the experimental group: the study by Mayer et al. [22] on the use of recombinant activated factor VII for ICH, the study of Rockswold et al. [23] on the use of hyperbaric oxygen for TBI and the controversial study of Cruz et al. [24] on the use of high dose mannitol for TBI with mydriasis. Unfortunately, the benefit of recombinant activated factor VII for ICH could not be confirmed in two later trials and both trials on TBI were not devoid of the risk of bias. The study of Cruz et al. is subject to ongoing scientific debate due to suspicion of data manipulation or fabrication [24]. However, the use of mannitol is widely established and represents a slightly different concept than blocking a metabolic pathway or suppressing an active reaction of the organism. Patients with TBI entered into the trial were on the verge of brain herniation due to cerebral edema and high dose mannitol helped the physiological measures of compensation. In this context the recently published RESCUE-ICP trial using decompressive craniectomy provided similar results [38]. Nonetheless, based on all the trials it appears doubtful that addressing SBD can be maintained as a generally valid treatment concept for ischemic stroke, TBI and ICH.

For aSAH the pooled risk ratio for poor outcome in the experimental groups was with 0.94 clearly below 1. A study on nimodipine showed a statistically significant benefit and the subgroup analysis showed a tendency towards a beneficial effect for the effector mechanism category “oxidative metabolism/stress”. Therefore it can be assumed that the concept of SBD as a therapeutic target has some value for the management of aSAH in the current setting.

Why does aSAH differ from ischemic stroke, TBI and ICH? A possible explanation could be that during the natural course after aSAH secondary processes are well balanced to allow for the best possible recovery, i.e. minimizing the chance of rebleed and ischemic damage by vasospasm. The data of the Cooperative Study [39] showed that during the natural course after aSAH rebleeds were less frequent in case of vasospasm, in other words that vasospasm protected to some degree against re-rupture. Since the introduction of early aneurysm elimination, the protective effect of vasospasm is no longer needed and only the negative side effects remain, i.e. delayed cerebral ischemia. Therefore, in the case of aSAH and eliminated source of bleeding, blocking physiological reactions might make some sense.

Methylprednisolone showed a marginally significant benefit for aSAH in one study [21], while corticosteroids were shown to be ineffective or even toxic for TBI and ICH. In the light of the above remarks regarding calcium antagonists it appears that corticosteroids likely benefit patients after aSAH, but corticosteroids have not been appropriately evaluated for aSAH.

Dexamethasone was shown in a number of, although marginally powered, studies to reduce the chance of death or poor outcome in patients with bacterial meningitis. The setting of meningitis is interesting. Similar to aSAH, the current setting with antibiotic treatment differ widely from the natural course and the natural secondary reactions of the immune system are not optimally adapted to the current situation, or in other words, the natural defense and clean-up mechanisms may become activated more than necessary under antibiotic treatment and therefore lead to unnecessary harm.

While the actual analysis could confirm a beneficial effect of pharmacological treatment of SBD only for aSAH and intracranial infection, cohort and population-based studies have clearly demonstrated a distinct reduction of overall mortality across all stroke entities and even TBI over the past decades [32, 38, 39]. In view of the absent or very limited effect of SBD directed treatments for ischemic stroke, ICH and TBI, it must be concluded that the positive time-trends during the last decades were mainly achieved by more aggressive early management and improved general measures of critical care [40]. Furthermore, treatments targeting the primary cause of brain damage, e.g. thrombectomy for ischemic stroke, seem to be generally more efficacious than studies focusing on sole neuroprotection or targeted reduction of specific SBD mechanisms [41–46]. It must be assumed that the extent of primary brain injury per se is by far the dominant prognostic factor. This causality seems to be especially valid for aSAH and ICH but also for ischemic stroke or TBI [44–50].

Our study has several limitations: First, a pooled analysis of treatment effects among different entities may certainly be controversial due to heterogeneous studies or therapeutic interventions per se. Nevertheless, the aim of this study was to illustrate the status quo of the overall effect of specific treatment of SBD for each entity and to highlight some potential explanations. Second, we primarily focused on death as a robust outcome event, since there were heterogeneous definitions for poor functional outcome in the majority of the underlying trials. However, both of these endpoints are not specific for SBD. We accounted for this by excluding trials that target both primary and secondary causes of brain injury. We cannot state with absolute certainty that we did not exclude by this an effective treatment concept actually working on SBD rather than the primary cause. Moreover, variations in outcome severity distribution might be inherent to the underlying conditions: While the mortality rate in patients undergoing endovascular thrombectomy for large-vessel stroke is 15%, the mortality for aSAH can be as high as 44% and for TBI as high as 72%, depending on regional or age-related factors, respectively [51–53]. Accordingly, in conditions with a relatively small proportion of fatal and poor outcomes, our approach might underestimate relatively subtle treatment effects on good or excellent outcomes. Third, with regard to the validity of SBD as a useful target of treatment we cannot exclude that the modern generally accepted critical care concepts also used within the clinical trials contributed to the general reduction of SBD [7–11, 54]. This may partially explain why more specific or targeted experimental treatments have failed to additionally improve outcome. However, methodological problems, such as imbalances in major baseline variables or prognostic factors, insufficient patient numbers as well as errors, noise or inconsistencies in outcome assessment, offer an alternative explanation why studies on SBD might fail to detect moderate treatment effects that could nevertheless be clinically relevant. This issue could be addressed by methodological improvements of future randomized trials, but also by focusing more on common data elements to facilitate meta-analyses, or even by contributing individual patient data for pooled analyses [55–57]. Additionally, assuming that SBD is a useful target of treatment, negative results of a methodologically sound trial might be related to the compound being tested, e.g. its effectiveness for the intended mechanism, or the pharmacokinetics and pharmacodynamics, e.g. dosing and timing. Finally, the strict selection criteria of included trials (only randomized, controlled trials) might have somewhat biased the results due to exclusion of some larger non-randomized observational studies.

## Conclusions

In summary, our meta-analysis on targeted treatment of specific SBD mechanisms following acute cerebral injury showed that a few selected SBD directed medications are likely to reduce the rate of poor outcome and death following aSAH, bacterial meningitis and maybe TBI, while no evidence for the usefulness of SBD directed medications was found in ischemic stroke and ICH. The result should lead to a new perspective of secondary reactions following cerebral injury. These processes should not be seen as suicide mechanisms that need to be fought. They should be rather seen as well orchestrated clean-up mechanisms that may today be somewhat too active in a few very specific constellations, such as meningitis under antibiotic treatment and aSAH after aneurysm elimination. Furthermore, these results should stimulate increased focus on methodological and reporting aspects of future trials.

## Additional file

Additional file 1: Risk of bias assessment and references to all studies enrolled in the current meta-analysis: This additional file contains supplementary information on the risk of bias assessment (allocation concealment and blinding), study treatments and patient numbers in treatment and control arms. Additionally, references to all studies enrolled in this meta-analysis are presented. (DOC 265 kb)

## Abbreviations

aSAH: aneurysmal subarachnoid hemorrhage
BI: Barthel Index
CI: Confidence Interval
GOS: Glasgow Outcome Scale
ICH: Intracerebral hemorrhage
mRS: modified Rankin Scale (mRS)
RR: Risk ratio
SBD: Secondary brain damage
TBI: Traumatic brain injury
