# Supplementary material for: Pharmacological targeting of secondary brain damage following ischemic or hemorrhagic stroke, traumatic brain injury, and bacterial meningitis - a systematic review and meta-analysis
Source: BMC Neurol. 2017 Dec 7;17:209. doi: 10.1186/s12883-017-0994-z (PMC5719738; doi:10.1186/s12883-017-0994-z)
Supplement: Additional file 1: — Risk of bias assessment and references to all studies enrolled in the current meta-analysis: This additional file contains supplementary information on the risk of bias assessment (allocation concealment and blinding), study treatments and patient numbers in treatment and control arms. Additionally, references to all studies enrolled in this meta-analysis are presented. (DOC 265 kb) [file 12883_2017_994_MOESM1_ESM.doc]

**Supplementary Information Table 1**

Risk of bias assessment for ischemic stroke meta-analysis.

| **First author** | **Year** | | **Allocation concealment** | | **Blinding** | | **Treatment** | | **No. Pat. Total** | | **No. Pat. Treatment** | | **No. Pat. Control** | |
| --- | --- | --- | --- | --- | --- | --- | --- | --- | --- | --- | --- | --- | --- | --- |
| Albers | | 2001 | | unclear | | yes | | aptiganel hydrochloride | | 628 | | 414 | | 214 |
| American Nimodipine Study Group | | 1992 | | unclear | | unclear | | nimodipine | | 1064 | | 800 | | 264 |
| Bogousslavsky | | 2002 | | unclear | | yes | | trafermin | | 287 | | 184 | | 106 |
| Chamorro | | 2014 | | yes | | yes | | uric acid | | 411 | | 211 | | 200 |
| Clark | | 2000 | | yes | | yes | | cervene/nalmefene | | 368 | | 163 | | 167 |
| De Deyn | | 1997 | | yes | | yes | | piracetam | | 927 | | 464 | | 463 |
| Den Hertog | | 2009 | | no | | yes | | paracetamol | | 1400 | | 697 | | 703 |
| Diener | | 2000 | | unclear | | yes | | lubeluzole | | 1813 | | 885 | | 901 |
| Diener | | 2008 | | yes | | yes | | DP-b99 | | 150 | | 75 | | 75 |
| Diener | | 2002 | | yes | | yes | | AR-R15896AR | | 103 | | 70 | | 33 |
| Ehrenreich | | 2002 | | unclear | | yes | | recombinant EPO | | 40 | | 21 | | 19 |
| Ehrenreich | | 2009 | | yes | | yes | | recombinant EPO | | 522 | | 256 | | 266 |
| Ginsberg | | 2013 | | yes | | yes | | albumin | | 841 | | 422 | | 419 |
| Grotta | | 1997 | | unclear | | yes | | lubeluzole | | 721 | | 368 | | 353 |
| Grotta | | 2001 | | yes | | yes | | lubeluzole | | 89 | | 45 | | 44 |
| He | | 2011 | | yes | | yes | | sanchitongtshu | | 145 | | 71 | | 69 |
| Horn | | 2001 | | unclear | | yes | | nimodipine | | 454 | | 225 | | 229 |
| Hsu | | 1988 | | unclear | | unclear | | pentoxifylline | | 297 | | 139 | | 131 |
| Hsu | | 1987 | | unclear | | unclear | | prostacyclin | | 80 | | 43 | | 37 |
| Huczynski | | 1985 | | unclear | | unclear | | prostacyclin | | 26 | | 13 | | 13 |
| IMAGES study investigators | | 2004 | | yes | | yes | | magnesium sulfate | | 2589 | | 1292 | | 1297 |
| Imai | | 2006 | | unclear | | no | | hyperbaric oxygen + edaravone | | 38 | | 19 | | 19 |
| Krams | | 2003 | | yes | | yes | | UK-279,276 | | 966 | | 718 | | 248 |
| Ladurner | | 2005 | | yes | | yes | | cerebrolysin | | 146 | | 78 | | 68 |
| Lees | | 2006 | | yes | | yes | | NYX-059 | | 1699 | | 858 | | 847 |
| Lees | | 2000 | | yes | | yes | | gavestinel | | 1804 | | 891 | | 897 |
| Lees | | 2013 | | yes | | yes | | DP-b99 | | 437 | | 218 | | 219 |
| Lodder | | 2006 | | no | | yes | | diazepam | | 748 | | 380 | | 368 |
| Lyden | | 2002 | | yes | | yes | | chlomethiazole | | 1198 | | 599 | | 599 |
| Martinez-Villa | | 1990 | | unclear | | unclear | | nimodipine | | 164 | | 81 | | 83 |
| Martinsson | | 2003 | | unclear | | unclear | | dexamphetamine | | 45 | | 30 | | 15 |
| Montaner | | 2008 | | unclear | | yes | | simvastatin | | 60 | | 30 | | 30 |
| Muir | | 2000 | | unclear | | yes | | sipatrigine | | 27 | | 21 | | 6 |
| Muir | | 1995 | | yes | | yes | | magnesium sulfate | | 60 | | 30 | | 30 |
| Muscari | | 2001 | | unclear | | unclear | | atorvastatin | | 62 | | 31 | | 31 |
| Oczkowski | | 1989 | | unclear | | unclear | | PY108-068 | | 19 | | 9 | | 10 |
| Ringelstein | | 2013 | | yes | | yes | | G-CSF | | 324 | | 161 | | 163 |
| Sandset | | 2011 | | yes | | yes | | candesartan | | 2029 | | 1017 | | 1012 |
| Saver | | 2015 | | unclear | | unclear | | magnesium | | 1700 | | 857 | | 843 |
| Schrader | | 2003 | | unclear | | unclear | | candesartan | | 342 | | 175 | | 187 |
| Shibuya | | 2005 | | no | | yes | | fasudil | | 160 | | 81 | | 79 |
| Shuaib | | 2007 | | yes | | yes | | NXY-059 | | 3195 | | 1588 | | 1607 |
| Teal | | 2009 | | yes | | yes | | repinotan | | 681 | | 342 | | 337 |
| Wahlgren | | 1999 | | unclear | | yes | | clomethiazole | | 1360 | | 680 | | 680 |
| Yamaguchi | | 1998 | | no | | unclear | | ebselen | | 302 | | 152 | | 150 |

“Year” refers to year of publication. According to the Cochrane Collaboration’s guidelines, “allocation concealment” contains reviewers’ judgment of whether allocation could have been foreseen prior to or during enrolment. “Blinding” refers to the reviewers’ judgment of adequate prevention of treatment allocation during the study. Judgment was either “yes” (i.e. low risk of bias), “no” (i.e. high risk of bias) or “unclear” (i.e. uncertain risk of bias).

**Supplementary Information Table 2**

Risk of bias assessment for aneurysmal subarachnoid hemorrhage meta-analysis.

| **First author** | **Year** | | **Allocation concealment** | | **Blinding** | | **Treatment** | | **No. Pat. Total** | | **No. Pat. Treatment** | | **No. Pat. Control** | |
| --- | --- | --- | --- | --- | --- | --- | --- | --- | --- | --- | --- | --- | --- | --- |
| Allen | | 1983 | | unclear | | unclear | | nimodipine | | 116 | | 56 | | 60 |
| Asano | | 1996 | | yes | | yes | | AVS (hydroxyl radical scavenger) | | 162 | | 80 | | 82 |
| Bradford | | 2013 | | yes | | no | | magnesium | | 162 | | 81 | | 81 |
| Chou | | 2008 | | unclear | | unclear | | simvastatin | | 30 | | 20 | | 19 |
| Dorhout Mees | | 2012 | | yes | | yes | | magnesium | | 1203 | | 606 | | 597 |
| Etminan | | 2013 | | yes | | no | | intraventricular fibrinolysis and head motion therapy | | 60 | | 30 | | 30 |
| Findlay | | 1995 | | unclear | | yes | | intracisternal tissue plasminogen activator | | 100 | | 51 | | 49 |
| Gomis | | 2010 | | unclear | | unclear | | methylprednisolone | | 95 | | 49 | | 46 |
| Haley | | 1995 | | unclear | | unclear | | tirilazad | | 245 | | 184 | | 61 |
| Haley | | 1997 | | unclear | | unclear | | tirilazad | | 897 | | 597 | | 300 |
| Haley | | 1993 | | yes | | yes | | nicardipine | | 886 | | 438 | | 448 |
| Hop | | 2000 | | unclear | | unclear | | aspirin | | 50 | | 24 | | 26 |
| Kassell | | 1996 | | unclear | | unclear | | tirilazad | | 1015 | | 762 | | 253 |
| Kirkpatrick | | 2014 | | yes | | yes | | simvastatin | | 803 | | 391 | | 412 |
| Lanzino | | 1999a | | unclear | | unclear | | tirilazad | | 817 | | 403 | | 414 |
| Lanzino | | 1999b | | unclear | | yes | | tirilazad | | 823 | | 410 | | 413 |
| Lynch | | 2005 | | unclear | | unclear | | simvastatin | | 39 | | 19 | | 20 |
| Macdonald | | 2008 | | unclear | | yes | | clazosentan | | 409 | | 313 | | 96 |
| Macdonald | | 2011 | | yes | | yes | | clazosentan | | 1157 | | 768 | | 389 |
| Macdonald | | 2012 | | yes | | yes | | clazosentan | | 571 | | 189 | | 382 |
| Neil-Dwyer | | 1987 | | unclear | | unclear | | nimodipine | | 75 | | 38 | | 37 |
| Öhman | | 1988 | | unclear | | unclear | | nimodipine | | 203 | | 104 | | 109 |
| Ohta | | 1986 | | unclear | | unclear | | nizofenone | | 208 | | 102 | | 106 |
| Ono | | 1984 | | unclear | | unclear | | ticlopidine | | 123 | | 65 | | 68 |
| Petruk | | 1988 | | yes | | yes | | nimodipine | | 154 | | 72 | | 82 |
| Pickard | | 1989 | | yes | | yes | | nimodipine | | 554 | | 278 | | 276 |
| Saito | | 1998 | | unclear | | yes | | ebselen | | 286 | | 145 | | 141 |
| Senbokuya | | 2013 | | yes | | yes | | cilostazol | | 109 | | 54 | | 55 |
| Shaw | | 2000 | | yes | | yes | | endothelin receptor antagonist | | 420 | | 213 | | 207 |
| Shibuya | | 1992 | | yes | | yes | | calcium antagonist | | 267 | | 131 | | 136 |
| Siironen | | 2003 | | unclear | | unclear | | enoxaparin | | 170 | | 85 | | 85 |
| Springborg | | 2007 | | yes | | yes | | erythropoietin | | 54 | | 24 | | 30 |
| Suzuki | | 1989 | | unclear | | unclear | | thromboxane synthetase inhibitor | | 256 | | 170 | | 86 |
| Tseng | | 2005 | | yes | | yes | | pravastatin | | 80 | | 40 | | 40 |
| Tseng | | 2009 | | yes | | yes | | erythropoietin | | 80 | | 40 | | 40 |
| Vajkoczy | | 2005 | | unclear | | yes | | clazosentan | | 32 | | 15 | | 17 |
| van den Bergh | | 2005 | | unclear | | unclear | | magnesium | | 283 | | 139 | | 144 |
| van den Bergh | | 2006 | | unclear | | unclear | | aspirin | | 161 | | 87 | | 74 |
| Vergouwen | | 2009 | | yes | | yes | | simvastatin | | 32 | | 16 | | 16 |
| Westermaier | | 2010 | | unclear | | unclear | | magnesium | | 107 | | 54 | | 53 |
| Wong | | 2010 | | yes | | yes | | magnesium | | 327 | | 169 | | 158 |

“Year” refers to year of publication. According to the Cochrane Collaboration’s guidelines, “allocation concealment” contains reviewers’ judgment of whether allocation could have been foreseen prior to or during enrolment. “Blinding” refers to the reviewers’ judgment of adequate prevention of treatment allocation during the study. Judgment was either “yes” (i.e. low risk of bias), “no” (i.e. high risk of bias) or “unclear” (i.e. uncertain risk of bias).

**Supplementary Information Table 3**

Risk of bias assessment for intracerebral hemorrhage meta-analysis.

| **First author** | **Year** | **Allocation concealment** | **Blinding** | **Treatment** | **No. Pat. Total** | **No. Pat. Treatment** | **No. Pat. Control** |
| --- | --- | --- | --- | --- | --- | --- | --- |
| Haley | 2005 | unclear | unclear | gavestinel | 565 | 280 | 285 |
| Lyden | 2007 | yes | yes | NXY-059 | 603 | 300 | 303 |
| Mayer | 2006 | unclear | unclear | factor VII | 40 | 32 | 8 |
| Mayer | 2008 | unclear | unclear | factor VII | 841 | 573 | 268 |
| Mayer | 2005 | yes | yes | factor VII | 399 | 303 | 96 |
| Misra | 2005 | unclear | unclear | mannitol | 128 | 65 | 63 |
| Poungvarin | 1987 | unclear | unclear | dexamethasone | 93 | 46 | 47 |
| Secades | 2006 | yes | yes | citicoline | 38 | 19 | 19 |
| Wahlgren | 2000 | unclear | unclear | clomethiazole | 94 | 47 | 47 |

“Year” refers to year of publication. According to the Cochrane Collaboration’s guidelines, “allocation concealment” contains reviewers’ judgment of whether allocation could have been foreseen prior to or during enrolment. “Blinding” refers to the reviewers’ judgment of adequate prevention of treatment allocation during the study. Judgment was either “yes” (i.e. low risk of bias), “no” (i.e. high risk of bias) or “unclear” (i.e. uncertain risk of bias).

**Supplementary Information Table 4**

Risk of bias assessment for traumatic brain injury meta-analysis.

| **First author** | **Year** | | **Allocation concealment** | | **Blinding** | | **Treatment** | | **No. Pat. Total** | | **No. Pat. Treatment** | | **No. Pat. Control** | |
| --- | --- | --- | --- | --- | --- | --- | --- | --- | --- | --- | --- | --- | --- | --- |
| Braakman | | 1983 | | yes | | yes | | dexamethasone | | 161 | | 81 | | 80 |
| Coester | | 2010 | | yes | | no | | intensive insulin therapy | | 88 | | 42 | | 46 |
| Cooper | | 2004 | | yes | | yes | | hypertonic saline resuscitation | | 229 | | 114 | | 115 |
| CRASH Trial Collaborators | | 2004 | | yes | | yes | | methylprednisolone | | 9964 | | 4985 | | 4979 |
| Cruz | | 2004 | | no | | no | | mannitol | | 44 | | 23 | | 21 |
| European Study Group on Nimodipine | | 1994 | | unclear | | yes | | nimodipine | | 819 | | 405 | | 414 |
| Haltiner | | 1999 | | unclear | | no | | phenytoin | | 404 | | 208 | | 196 |
| Hatton | | 2008 | | unclear | | unclear | | cyclosporine | | 40 | | 32 | | 8 |
| Marshall | | 1998 | | unclear | | yes | | tirilazad mesylate | | 1120 | | 562 | | 558 |
| Morris | | 1999 | | unclear | | yes | | selfotel | | 693 | | 324 | | 331 |
| Muizelaar | | 1991 | | unclear | | no | | hyperventilation + tromethamine | | 113 | | 72 | | 41 |
| Muizelaar | | 1993 | | unclear | | unclear | | superoxide dismutase | | 104 | | 78 | | 26 |
| Rockswold | | 1992 | | unclear | | no | | hyperbaric oxygen | | 168 | | 84 | | 84 |
| Rockswold | | 2013 | | unclear | | no | | hyperoxia | | 42 | | 20 | | 22 |
| SAFE Investigators | | 2007 | | yes | | yes | | albumin | | 460 | | 231 | | 229 |
| Sakellaris | | 2006 | | unclear | | unclear | | creatine | | 39 | | 20 | | 19 |
| Saul | | 1981 | | unclear | | unclear | | methylprednisolone | | 100 | | 50 | | 50 |
| Skolnick | | 2014 | | yes | | yes | | progesterone | | 1179 | | 591 | | 588 |
| Temkin | | 2007 | | yes | | yes | | magnesium | | 447 | | 225 | | 222 |
| Wright | | 2014 | | yes | | yes | | progesterone | | 882 | | 442 | | 440 |

“Year” refers to year of publication. According to the Cochrane Collaboration’s guidelines, “allocation concealment” contains reviewers’ judgment of whether allocation could have been foreseen prior to or during enrolment. “Blinding” refers to the reviewers’ judgment of adequate prevention of treatment allocation during the study. Judgment was either “yes” (i.e. low risk of bias), “no” (i.e. high risk of bias) or “unclear” (i.e. uncertain risk of bias).

**Supplementary Information Table 5**

Risk of bias assessment bacterial meningitis meta-analysis.

| **First author** | **Year** | | **Allocation concealment** | | **Blinding** | | **Treatment** | | **No. Pat. Total** | | **No. Pat. Treatment** | | **No. Pat. Control** | |
| --- | --- | --- | --- | --- | --- | --- | --- | --- | --- | --- | --- | --- | --- | --- |
| De Gans | | 2002 | | yes | | yes | | dexamethasone | | 301 | | 157 | | 144 |
| Mathur | | 2013 | | yes | | no | | dexamethasone | | 80 | | 40 | | 40 |
| Molyneux | | 2002 | | unclear | | yes | | dexamethasone | | 598 | | 307 | | 295 |
| Nguyen | | 2007 | | unclear | | yes | | dexamethasone | | 435 | | 217 | | 218 |
| Odio | | 1991 | | unclear | | yes | | dexamethasone | | 101 | | 52 | | 49 |
| Quazi | | 1996 | | unclear | | yes | | dexamethasone | | 89 | | 48 | | 41 |
| Scarborough | | 2007 | | unclear | | unclear | | dexamethasone | | 465 | | 233 | | 232 |
| Thwaites | | 2004 | | no | | yes | | dexamethasone | | 545 | | 274 | | 271 |

“Year” refers to year of publication. According to the Cochrane Collaboration’s guidelines, “allocation concealment” contains reviewers’ judgment of whether allocation could have been foreseen prior to or during enrolment. “Blinding” refers to the reviewers’ judgment of adequate prevention of treatment allocation during the study. Judgment was either “yes” (i.e. low risk of bias), “no” (i.e. high risk of bias) or “unclear” (i.e. uncertain risk of bias).

**References for ischemic stroke meta-analysis**

1. Albers GW, Goldstein LB, Hall D, et al. Aptiganel hydrochloride in acute ischemic stroke: a randomized controlled trial. JAMA 2001; 286:2673-82. http://www.ncbi.nlm.nih.gov/pubmed/11730442 (accessed 10 Aug2014).
2. American Nimodipin Study Group. Clinical trial of nimodipine in acute ischemic stroke. The American Nimodipine Study Group. Stroke 1992;23:3–8.http://www.ncbi.nlm.nih.gov/pubmed/1731418 (accessed 10 Aug2014).
3. Bogousslavsky J, Victor SJ, Salinas EO, et al. Fiblast (Trafermin) in Acute Stroke: Results of the European-Australian Phase II/III Safety and Efficacy Trial. Cerebrovasc Dis 2002;14:239–51. doi:10.1159/000065683
4. [Chamorro A](http://www.ncbi.nlm.nih.gov/pubmed/?term=Chamorro A%5BAuthor%5D&cauthor=true&cauthor_uid=24703208), [Amaro S](http://www.ncbi.nlm.nih.gov/pubmed/?term=Amaro S%5BAuthor%5D&cauthor=true&cauthor_uid=24703208), [Castellanos M](http://www.ncbi.nlm.nih.gov/pubmed/?term=Castellanos M%5BAuthor%5D&cauthor=true&cauthor_uid=24703208), et al. Safety and efficacy of uric acid in patients with acute stroke (URICO-ICTUS): a randomised, double-blind phase 2b/3 trial. [Lancet Neurol.](http://www.ncbi.nlm.nih.gov/pubmed/24703208) 2014 May;13(5):453-60. doi: 10.1016/S1474-4422(14)70054-7. Epub 2014 Apr 2.
5. Clark WM, Raps EC, Tong DC, et al. Cervene (Nalmefene) in Acute Ischemic Stroke : Final Results of a Phase III Efficacy Study. Stroke 2000;31:1234–9. doi:10.1161/01.STR.31.6.1234
6. De Deyn PP, De Reuck J, Deberdt W, et al. Treatment of Acute Ischemic Stroke With Piracetam. Stroke 1997;28:2347–52. doi:10.1161/01.STR.28.12.2347
7. den Hertog HM, van der Worp HB, van Gemert HM a, et al. The Paracetamol (Acetaminophen) In Stroke (PAIS) trial: a multicentre, randomised, placebo-controlled, phase III trial. Lancet Neurol 2009;8:434–40. doi:10.1016/S1474-4422(09)70051-1
8. Diener HC, Cortens M, Ford G, et al. Lubeluzole in Acute Ischemic Stroke Treatment : A Double-Blind Study With an 8-Hour Inclusion Window Comparing a 10-mg Daily Dose of Lubeluzole With Placebo. Stroke 2000;31:2543–51. doi:10.1161/01.STR.31.11.2543
9. Diener H-C, AlKhedr A, Busse O, et al. Treatment of acute ischaemic stroke with the low-affinity, use-dependent NMDA antagonist AR-R15896AR. A safety and tolerability study. J Neurol 2002;249:561–8. doi:10.1007/s004150200065
10. Diener H-C, Schneider D, Lampl Y, et al. DP-b99, a membrane-activated metal ion chelator, as neuroprotective therapy in ischemic stroke. Stroke 2008;39:1774–8. doi:10.1161/STROKEAHA.107.506378
11. Ehrenreich H, Hasselblatt M, Dembowski C, et al. Clinical Trial Erythropoietin Therapy for Acute Stroke Is Both Safe and Beneficial. 2002;8:495–505.
12. Ehrenreich H, Weissenborn K, Prange H, et al. Recombinant human erythropoietin in the treatment of acute ischemic stroke. Stroke 2009;40:e647–56. doi:10.1161/STROKEAHA.109.564872
13. Ginsberg MD, Palesch YY, Hill MD, et al. High-dose albumin treatment for acute ischaemic stroke (ALIAS) Part 2: a randomised, double-blind, phase 3, placebo-controlled trial. Lancet Neurol 2013;12:1049–58. doi:10.1016/S1474-4422(13)70223-0
14. Grotta J. Combination Therapy Stroke Trial: recombinant tissue-type plasminogen activator with/without lubeluzole. Cerebrovasc Dis 2001;12:258–63. doi:47713
15. Grotta J. Lubeluzole treatment of acute ischemic stroke. The US and Canadian Lubeluzole Ischemic Stroke Study Group. Stroke 1997;28:2338–46.http://www.ncbi.nlm.nih.gov/pubmed/9412611 (accessed 26 Sep2014).
16. He L, Chen X, Zhou M, et al. Radix/rhizoma notoginseng extract (sanchitongtshu) for ischemic stroke: a randomized controlled study. Phytomedicine 2011;18:437–42. doi:10.1016/j.phymed.2010.10.004
17. Horn J, de Haan RJ, Vermeulen M, et al. Very Early Nimodipine Use in Stroke (VENUS) : A Randomized, Double-Blind, Placebo-Controlled Trial. Stroke 2001;32:461–5. doi:10.1161/01.STR.32.2.461
18. Hsu CY, Faught RE, Furlan a. J, et al. Intravenous prostacyclin in acute nonhemorrhagic stroke: a placebo- controlled double-blind trial. Stroke 1987;18:352–8. doi:10.1161/01.STR.18.2.352
19. Hsu CY, Norris JW, Hogan EL, et al. Pentoxifylline in acute nonhemorrhagic stroke. A randomized, placebo- controlled double-blind trial. Stroke 1988;19:716–22. doi:10.1161/01.STR.19.6.716
20. Huczynski J, Kostka-Trabka E, Sotowska W, et al. Double-blind controlled trial of the therapeutic effects of prostacyclin in patients with completed ischaemic stroke. Stroke 1985;16:810–4. doi:10.1161/01.STR.16.5.810
21. Imai K, Mori T, Izumoto H, et al. Hyperbaric oxygen combined with intravenous edaravone for treatment of acute embolic stroke: a pilot clinical trial. Neurol Med Chir (Tokyo) 2006;46:373–8; discussion 378.http://www.ncbi.nlm.nih.gov/pubmed/16936457
22. Krams M, Lees KR, Hacke W, et al. Acute Stroke Therapy by Inhibition of Neutrophils (ASTIN): an adaptive dose-response study of UK-279,276 in acute ischemic stroke. Stroke 2003;34:2543–8. doi:10.1161/01.STR.0000092527.33910.89
23. Ladurner G, Kalvach P, Moessler H. Neuroprotective treatment with cerebrolysin in patients with acute stroke: a randomised controlled trial. J Neural Transm 2005;112:415–28. doi:10.1007/s00702-004-0248-2
24. Lees K, Asplund K, Carolei a, et al. Glycine antagonist (gavestinel) in neuroprotection (GAIN International) in patients with acute stroke: a randomised controlled trial. Lancet 2000;355:1949–54. doi:10.1016/S0140-6736(00)02326-6
25. Lees KR, Bornstein N, Diener H-C, et al. Results of Membrane-Activated Chelator Stroke Intervention randomized trial of DP-b99 in acute ischemic stroke. Stroke 2013;44:580–4. doi:10.1161/STROKEAHA.111.000013
26. Lees KR, Zivin J a, Ashwood T, et al. NXY-059 for acute ischemic stroke. N Engl J Med 2006;354:588–600. doi:10.1056/NEJMoa052980
27. Lodder J, van Raak L, Hilton a, et al. Diazepam to improve acute stroke outcome: results of the early GABA-Ergic activation study in stroke trial. a randomized double-blind placebo-controlled trial. Cerebrovasc Dis 2006;21:120–7. doi:10.1159/000090210
28. Lyden P. Clomethiazole Acute Stroke Study in Ischemic Stroke (CLASS-I): Final Results * Editorial Comment: Final Results. Stroke 2002;33:122–9. doi:10.1161/hs0102.101478
29. Martinez-Vila E, Guillen F, Villanueva J a., et al. Placebo-controlled trial of nimodipine in the treatment of acute ischemic cerebral infarction. Stroke 1990;21:1023–8. doi:10.1161/01.STR.21.7.1023
30. Martinsson L. Safety of Dexamphetamine in Acute Ischemic Stroke: A Randomized, Double-Blind, Controlled Dose-Escalation Trial. Stroke 2002;34:475–81. doi:10.1161/01.STR.0000050161.38263.AE
31. Montaner J, Chacón P, Krupinski J, et al. Simvastatin in the acute phase of ischemic stroke: a safety and efficacy pilot trial. Eur J Neurol 2008;15:82–90. doi:10.1111/j.1468-1331.2007.02015.x
32. Muir KW, Holzapfel L, Lees KR. Phase II clinical trial of sipatrigine (619C89) by continuous infusion in acute stroke. Cerebrovasc Dis 2000;10:431–6. doi:16103
33. Muir KW, Lees KR. A randomized, double-blind, placebo-controlled pilot trial of intravenous magnesium sulfate in acute stroke. Ann N Y Acad Sci 1995;765:315–6.http://www.ncbi.nlm.nih.gov/pubmed/7486627
34. Muir KW, Lees KR, Ford I, et al. Magnesium for acute stroke (Intravenous Magnesium Efficacy in Stroke trial): randomised controlled trial. Lancet 2004;363:439–45. doi:10.1016/S0140-6736(04)15490-1
35. Muscari A, Puddu GM, Santoro N, et al. The atorvastatin during ischemic stroke study: a pilot randomized controlled trial. Clin Neuropharmacol 2011;34:141–7. doi:10.1097/WNF.0b013e3182206c2f
36. Oczkowski WJ, Hachinski VC, Bogousslavsky J, et al. A double-blind, randomized trial of PY108-068 in acute ischemic cerebral infarction. Stroke 1989;20:604–8. doi:10.1161/01.STR.20.5.604
37. Ringelstein EB, Thijs V, Norrving B, et al. Granulocyte colony-stimulating factor in patients with acute ischemic stroke: results of the AX200 for Ischemic Stroke trial. Stroke 2013;44:2681–7. doi:10.1161/STROKEAHA.113.001531
38. Sandset EC, Bath PMW, Boysen G, et al. The angiotensin-receptor blocker candesartan for treatment of acute stroke (SCAST): a randomised, placebo-controlled, double-blind trial. Lancet 2011;377:741–50. doi:10.1016/S0140-6736(11)60104-9
39. Saver JL, Starkman S, Eckstein M, et al. [Prehospital use of magnesium sulfate as neuroprotection in acute stroke.](http://www.ncbi.nlm.nih.gov/pubmed/25651247) N Engl J Med. 2015 Feb 5;372(6):528-36. doi: 10.1056/NEJMoa1408827
40. Schrader J, Lüders S, Kulschewski A, et al. The ACCESS Study: evaluation of Acute Candesartan Cilexetil Therapy in Stroke Survivors. Stroke 2003;34:1699–703. doi:10.1161/01.STR.0000075777.18006.89
41. Shibuya M, Hirai S, Seto M, et al. Effects of fasudil in acute ischemic stroke: results of a prospective placebo-controlled double-blind trial. J Neurol Sci 2005;238:31–9. doi:10.1016/j.jns.2005.06.003
42. Shuaib A, Lees KR, Lyden P, et al. NXY-059 for the treatment of acute ischemic stroke. N Engl J Med 2007;357:562–71. doi:10.1056/NEJMoa070240
43. Teal P, Davis S, Hacke W, et al. A randomized, double-blind, placebo-controlled trial to evaluate the efficacy, safety, tolerability, and pharmacokinetic/pharmacodynamic effects of a targeted exposure of intravenous repinotan in patients with acute ischemic stroke: modified Randomized Ex. Stroke 2009;40:3518–25. doi:10.1161/STROKEAHA.109.551382
44. Wahlgren NG, Ranasinha KW, Rosolacci T, et al. Clomethiazole Acute Stroke Study (CLASS) : Results of a Randomized, Controlled Trial of Clomethiazole Versus Placebo in 1360 Acute Stroke Patients. Stroke 1999;30:21–8. doi:10.1161/01.STR.30.1.21
45. Yamaguchi T, Sano K, Takakura K, et al. Ebselen in Acute Ischemic Stroke : A Placebo-Controlled, Double-blind Clinical Trial. Stroke 1998;29:12–7. doi:10.1161/01.STR.29.1.12

**References for aneurysmal subarachnoid hemorrhage meta-analysis**

1. Allen GS, Ahn HS, Preziosi TJ, et al. Cerebral arterial spasm--a controlled trial of nimodipine in patients with subarachnoid hemorrhage. N Engl J Med 1983;308:619–24. doi:10.1056/NEJM198303173081103
2. Asano T, Takakura K, Sano K, et al. Effects of a hydroxyl radical scavenger on delayed ischemic neurological deficits following aneurysmal subarachnoid hemorrhage: results of a multicenter, placebo-controlled double-blind trial. J Neurosurg 1996;84:792–803. doi:10.3171/jns.1996.84.5.0792
3. Bradford CM, Finfer S, O’Connor A, et al. A randomised controlled trial of induced hypermagnesaemia following aneurysmal subarachnoid haemorrhage. Crit Care Resusc 2013;15:119–25.http://www.ncbi.nlm.nih.gov/pubmed/23931043
4. Chou SH-Y, Smith EE, Badjatia N, et al. A randomized, double-blind, placebo-controlled pilot study of simvastatin in aneurysmal subarachnoid hemorrhage. Stroke 2008;39:2891–3. doi:10.1161/STROKEAHA.107.505875
5. Dorhout Mees SM, Algra A, Vandertop WP, et al. Magnesium for aneurysmal subarachnoid haemorrhage (MASH-2): a randomised placebo-controlled trial. Lancet 2012;380:44–9. doi:10.1016/S0140-6736(12)60724-7
6. Etminan N, Beseoglu K, Eicker SO, et al. Prospective, randomized, open-label phase II trial on concomitant intraventricular fibrinolysis and low-frequency rotation after severe subarachnoid hemorrhage. Stroke 2013;44:2162–8. doi:10.1161/STROKEAHA.113.001790
7. Findlay JM, Kassell NF, Weir BK, et al. A randomized trial of intraoperative, intracisternal tissue plasminogen activator for the prevention of vasospasm. Neurosurgery 1995;37:168–76; discussion 177–8.http://www.ncbi.nlm.nih.gov/pubmed/8587685 (accessed 10 Aug2014).
8. Gomis P, Graftieaux JP, Sercombe R, et al. Randomized, double-blind, placebo-controlled, pilot trial of high-dose methylprednisolone in aneurysmal subarachnoid hemorrhage. J Neurosurg 2010;112:681–8. doi:10.3171/2009.4.JNS081377
9. Haley EC, Kassell NF, Alves WM, et al. Phase II trial of tirilazad in aneurysmal subarachnoid hemorrhage. A report of the Cooperative Aneurysm Study. J Neurosurg 1995;82:786–90. doi:10.3171/jns.1995.82.5.0786
10. Haley EC, Kassell NF, Apperson-Hansen C, et al. A randomized, double-blind, vehicle-controlled trial of tirilazad mesylate in patients with aneurysmal subarachnoid hemorrhage: a cooperative study in North America. J Neurosurg 1997;86:467–74. doi:10.3171/jns.1997.86.3.0467
11. Haley EC, Kassell NF, Torner JC. A randomized controlled trial of high-dose intravenous nicardipine in aneurysmal subarachnoid hemorrhage. A report of the Cooperative Aneurysm Study. J Neurosurg 1993;78:537–47. doi:10.3171/jns.1993.78.4.0537
12. Hop JW, Rinkel GJ, Algra A, et al. Randomized pilot trial of postoperative aspirin in subarachnoid hemorrhage. Neurology 2000;54:872–8.http://www.ncbi.nlm.nih.gov/pubmed/10690979 (accessed 10 Aug2014).
13. Kassell NF, Haley EC, Apperson-Hansen C, et al. Randomized, double-blind, vehicle-controlled trial of tirilazad mesylate in patients with aneurysmal subarachnoid hemorrhage: a cooperative study in Europe, Australia, and New Zealand. J Neurosurg 1996;84:221–8. doi:10.3171/jns.1996.84.2.0221
14. Kirkpatrick PJ, Turner CL, Smith C, et al. [Simvastatin in aneurysmal subarachnoid haemorrhage (STASH): a multicentre randomised phase 3 trial.](http://www.ncbi.nlm.nih.gov/pubmed/24837690) Lancet Neurol. 2014 Jul;13(7):666-75. doi: 10.1016/S1474-4422(14)70084-5. Epub 2014 May 15.
15. Lanzino G, Kassell NF. Double-blind, randomized, vehicle-controlled study of high-dose tirilazad mesylate in women with aneurysmal subarachnoid hemorrhage. Part II. A cooperative study in North America. J Neurosurg 1999;90:1018–24. doi:10.3171/jns.1999.90.6.1018
16. Lanzino G, Kassell NF, Dorsch NW, et al. Double-blind, randomized, vehicle-controlled study of high-dose tirilazad mesylate in women with aneurysmal subarachnoid hemorrhage. Part I. A cooperative study in Europe, Australia, New Zealand, and South Africa. J Neurosurg 1999;90:1011–7. doi:10.3171/jns.1999.90.6.1011
17. Lynch JR, Wang H, McGirt MJ, et al. Simvastatin reduces vasospasm after aneurysmal subarachnoid hemorrhage: results of a pilot randomized clinical trial. Stroke 2005;36:2024–6. doi:10.1161/01.STR.0000177879.11607.10
18. Macdonald RL, Higashida RT, Keller E, et al. Clazosentan, an endothelin receptor antagonist, in patients with aneurysmal subarachnoid haemorrhage undergoing surgical clipping: a randomised, double-blind, placebo-controlled phase 3 trial (CONSCIOUS-2). Lancet Neurol 2011;10:618–25. doi:10.1016/S1474-4422(11)70108-9
19. Macdonald RL, Higashida RT, Keller E, et al. Randomized trial of clazosentan in patients with aneurysmal subarachnoid hemorrhage undergoing endovascular coiling. Stroke 2012;43:1463–9. doi:10.1161/STROKEAHA.111.648980
20. Macdonald RL, Kassell NF, Mayer S, et al. Clazosentan to overcome neurological ischemia and infarction occurring after subarachnoid hemorrhage (CONSCIOUS-1): randomized, double-blind, placebo-controlled phase 2 dose-finding trial. Stroke 2008;39:3015–21. doi:10.1161/STROKEAHA.108.519942
21. Neil-Dwyer G, Mee E, Dorrance D, et al. Early intervention with nimodipine in subarachnoid haemorrhage. Eur Heart J 1987;8 Suppl K:41–7.http://www.ncbi.nlm.nih.gov/pubmed/3450521
22. Ohman J, Heiskanen O. Effect of nimodipine on the outcome of patients after aneurysmal subarachnoid hemorrhage and surgery. J Neurosurg 1988;69:683–6. doi:10.3171/jns.1988.69.5.0683
23. Ohta T, Kikuchi H, Hashi K, et al. Nizofenone administration in the acute stage following subarachnoid hemorrhage. Results of a multi-center controlled double-blind clinical study. J Neurosurg 1986;64:420–6. doi:10.3171/jns.1986.64.3.0420
24. Ono H, Mizukami M, Kitamura K, et al. Ticlopidine: quo vadis? Subarachnoid hemorrhage. Agents Actions Suppl 1984;15:259–72.http://www.ncbi.nlm.nih.gov/pubmed/6385650 (accessed 10 Aug2014).
25. Petruk KC, West M, Mohr G, et al. Nimodipine treatment in poor-grade aneurysm patients. Results of a multicenter double-blind placebo-controlled trial. J Neurosurg 1988;68:505–17. doi:10.3171/jns.1988.68.4.0505
26. Pickard JD, Murray GD, Illingworth R, et al. Effect of oral nimodipine on cerebral infarction and outcome after subarachnoid haemorrhage: British aneurysm nimodipine trial. BMJ 1989;298:636–42.http://www.pubmedcentral.nih.gov/articlerender.fcgi?artid=1835889&tool=pmcentrez&rendertype=abstract
27. Saito I, Asano T, Sano K, et al. Neuroprotective effect of an antioxidant, ebselen, in patients with delayed neurological deficits after aneurysmal subarachnoid hemorrhage. Neurosurgery 1998;42:269–77; discussion 277–8.http://www.ncbi.nlm.nih.gov/pubmed/9482177 (accessed 10 Aug2014).
28. Senbokuya N, Kinouchi H, Kanemaru K, et al. Effects of cilostazol on cerebral vasospasm after aneurysmal subarachnoid hemorrhage: a multicenter prospective, randomized, open-label blinded end point trial. J Neurosurg 2013;118:121–30. doi:10.3171/2012.9.JNS12492
29. Shaw MD, Vermeulen M, Murray GD, et al. Efficacy and safety of the endothelin, receptor antagonist TAK-044 in treating subarachnoid hemorrhage: a report by the Steering Committee on behalf of the UK/Netherlands/Eire TAK-044 Subarachnoid Haemorrhage Study Group. J Neurosurg 2000;93:992–7. doi:10.3171/jns.2000.93.6.0992
30. Shibuya M, Suzuki Y, Sugita K, et al. Effect of AT877 on cerebral vasospasm after aneurysmal subarachnoid hemorrhage. Results of a prospective placebo-controlled double-blind trial. J Neurosurg 1992;76:571–7. doi:10.3171/jns.1992.76.4.0571
31. Siironen J, Juvela S, Varis J, et al. No effect of enoxaparin on outcome of aneurysmal subarachnoid hemorrhage: a randomized, double-blind, placebo-controlled clinical trial. J Neurosurg 2003;99:953–9. doi:10.3171/jns.2003.99.6.0953
32. Springborg JB, Møller C, Gideon P, et al. Erythropoietin in patients with aneurysmal subarachnoid haemorrhage: a double blind randomised clinical trial. Acta Neurochir (Wien) 2007;149:1089–101; discussion 1101. doi:10.1007/s00701-007-1284-z
33. Suzuki S, Sano K, Handa H, et al. Clinical study of OKY-046, a thromboxane synthetase inhibitor, in prevention of cerebral vasospasms and delayed cerebral ischaemic symptoms after subarachnoid haemorrhage due to aneurysmal rupture: a randomized double-blind study. Neurol Res 1989;11:79–88.http://www.ncbi.nlm.nih.gov/pubmed/2569686 (accessed 10 Aug2014).
34. Tseng M-Y, Czosnyka M, Richards H, et al. Effects of acute treatment with pravastatin on cerebral vasospasm, autoregulation, and delayed ischemic deficits after aneurysmal subarachnoid hemorrhage: a phase II randomized placebo-controlled trial. Stroke 2005;36:1627–32. doi:10.1161/01.STR.0000176743.67564.5d
35. Tseng M-Y, Hutchinson PJ, Richards HK, et al. Acute systemic erythropoietin therapy to reduce delayed ischemic deficits following aneurysmal subarachnoid hemorrhage: a Phase II randomized, double-blind, placebo-controlled trial. Clinical article. J Neurosurg 2009;111:171–80. doi:10.3171/2009.3.JNS081332
36. Vajkoczy P, Meyer B, Weidauer S, et al. Clazosentan (AXV-034343), a selective endothelin A receptor antagonist, in the prevention of cerebral vasospasm following severe aneurysmal subarachnoid hemorrhage: results of a randomized, double-blind, placebo-controlled, multicenter phase IIa study. J Neurosurg 2005;103:9–17. doi:10.3171/jns.2005.103.1.0009
37. van den Bergh WM, Algra a, Dorhout Mees SM, et al. Randomized controlled trial of acetylsalicylic acid in aneurysmal subarachnoid hemorrhage: the MASH Study. Stroke 2006;37:2326–30. doi:10.1161/01.STR.0000236841.16055.0f
38. van den Bergh WM, Algra a, van Kooten F, et al. Magnesium sulfate in aneurysmal subarachnoid hemorrhage: a randomized controlled trial. Stroke 2005;36:1011–5. doi:10.1161/01.STR.0000160801.96998.57
39. Vergouwen MDI, Meijers JCM, Geskus RB, et al. Biologic effects of simvastatin in patients with aneurysmal subarachnoid hemorrhage: a double-blind, placebo-controlled randomized trial. J Cereb Blood Flow Metab 2009;29:1444–53. doi:10.1038/jcbfm.2009.59
40. Westermaier T, Stetter C, Vince GH, et al. Prophylactic intravenous magnesium sulfate for treatment of aneurysmal subarachnoid hemorrhage: a randomized, placebo-controlled, clinical study. Crit Care Med 2010;38:1284–90. doi:10.1097/CCM.0b013e3181d9da1e
41. Wong GKC, Poon WS, Chan MT V, et al. Intravenous magnesium sulphate for aneurysmal subarachnoid hemorrhage (IMASH): a randomized, double-blinded, placebo-controlled, multicenter phase III trial. Stroke 2010;41:921–6. doi:10.1161/STROKEAHA.109.571125

**References for intracerebral hemorrhage meta-analysis**

1. Haley EC, Thompson JLP, Levin B, et al. Gavestinel does not improve outcome after acute intracerebral hemorrhage: an analysis from the GAIN International and GAIN Americas studies. Stroke 2005;36:1006–10. doi:10.1161/01.STR.0000163053.77982.8d
2. Lyden PD, Shuaib A, Lees KR, et al. Safety and tolerability of NXY-059 for acute intracerebral hemorrhage: the CHANT Trial. Stroke 2007;38:2262–9. doi:10.1161/STROKEAHA.106.472746
3. Mayer SA, Brun NC, Begtrup K, et al. Recombinant activated factor VII for acute intracerebral hemorrhage. N Engl J Med 2005;352:777–85. doi:10.1056/NEJMoa042991
4. Mayer S a, Brun NC, Begtrup K, et al. Efficacy and safety of recombinant activated factor VII for acute intracerebral hemorrhage. N Engl J Med 2008;358:2127–37. doi:10.1056/NEJMoa0707534
5. Mayer SA, Brun NC, Broderick J, et al. Recombinant activated factor VII for acute intracerebral hemorrhage: US phase IIA trial. Neurocrit Care 2006;4:206–14. doi:10.1385/NCC:4:3:206
6. Misra UK, Kalita J, Ranjan P, et al. Mannitol in intracerebral hemorrhage: a randomized controlled study. J Neurol Sci 2005;234:41–5. doi:10.1016/j.jns.2005.03.038
7. Poungvarin N, Bhoopat W, Viriyavejakul a, et al. Effects of dexamethasone in primary supratentorial intracerebral hemorrhage. N Engl J Med 1987;316:1229–33. doi:10.1056/NEJM198705143162001
8. Secades JJ, Alvarez-Sabín J, Rubio F, et al. Citicoline in intracerebral haemorrhage: a double-blind, randomized, placebo-controlled, multi-centre pilot study. Cerebrovasc Dis 2006;21:380–5. doi:10.1159/000091547
9. Wahlgren NG, Diez-Tejedor E, Teitelbaum J, et al. Results in 95 Hemorrhagic Stroke Patients Included in CLASS, a Controlled Trial of Clomethiazole Versus Placebo in Acute Stroke Patients. Stroke 2000;31:82–5. doi:10.1161/01.STR.31.1.82

**References for traumatic brain injury meta-analysis**

1. Braakman R, Schouten HJ, Blaauw-van Dishoeck M, et al. Megadose steroids in severe head injury. Results of a prospective double-blind clinical trial. J Neurosurg 1983;58:326–30. doi:10.3171/jns.1983.58.3.0326
2. Coester A, Neumann CR, Schmidt MI. Intensive insulin therapy in severe traumatic brain injury: a randomized trial. J Trauma 2010;68:904–11. doi:10.1097/TA.0b013e3181c9afc2
3. Cooper DJ, Myles PS, McDermott FT, et al. Prehospital hypertonic saline resuscitation of patients with hypotension and severe traumatic brain injury: a randomized controlled trial. JAMA 2004;291:1350–7. doi:10.1001/jama.291.11.1350
4. CRASH trial collaborators. Effect of intravenous corticosteroids on death within 14 days in 10 008 adults with clinically significant head injury (MRC CRASH trial): Randomised placebo-controlled trial. Lancet 2004;364:1321–1328.
5. Cruz J, Minoja G, Okuchi K, et al. Successful use of the new high-dose mannitol treatment in patients with Glasgow Coma Scale scores of 3 and bilateral abnormal pupillary widening: a randomized trial. J Neurosurg 2004;100:376–83. doi:10.3171/jns.2004.100.3.0376
6. European Study Group on Nimodipine in Severe Head Injury. A multicenter trial of the efficacy of nimodipine on outcome after severe head injury. The European Study Group on Nimodipine in Severe Head Injury. J Neurosurg 1994;80:797–804. doi:10.3171/jns.1994.80.5.0797
7. Haltiner a M, Newell DW, Temkin NR, et al. Side effects and mortality associated with use of phenytoin for early posttraumatic seizure prophylaxis. J Neurosurg 1999;91:588–92. doi:10.3171/jns.1999.91.4.0588
8. Hatton J, Rosbolt B, Empey P, et al. Dosing and safety of cyclosporine in patients with severe brain injury. J Neurosurg 2008;109:699–707. doi:10.3171/JNS/2008/109/10/0699
9. Marshall LF, Maas a I, Marshall SB, et al. A multicenter trial on the efficacy of using tirilazad mesylate in cases of head injury. J Neurosurg 1998;89:519–25. doi:10.3171/jns.1998.89.4.0519
10. Morris GF, Bullock R, Marshall SB, et al. Failure of the competitive N-methyl-D-aspartate antagonist Selfotel (CGS 19755) in the treatment of severe head injury: results of two phase III clinical trials. The Selfotel Investigators. J Neurosurg 1999;91:737–43. doi:10.3171/jns.1999.91.5.0737
11. Muizelaar JP, Marmarou a, Ward JD, et al. Adverse effects of prolonged hyperventilation in patients with severe head injury: a randomized clinical trial. J Neurosurg 1991;75:731–9. doi:10.3171/jns.1991.75.5.0731
12. Muizelaar JP, Marmarou a, Young HF, et al. Improving the outcome of severe head injury with the oxygen radical scavenger polyethylene glycol-conjugated superoxide dismutase: a phase II trial. J Neurosurg 1993;78:375–82. doi:10.3171/jns.1993.78.3.0375
13. Rockswold GL, Ford SE, Anderson DC, et al. Results of a prospective randomized trial for treatment of severely brain-injured patients with hyperbaric oxygen. J Neurosurg 1992;76:929–34. doi:10.3171/jns.1992.76.6.0929
14. Rockswold SB, Rockswold GL, Zaun D a, et al. A prospective, randomized Phase II clinical trial to evaluate the effect of combined hyperbaric and normobaric hyperoxia on cerebral metabolism, intracranial pressure, oxygen toxicity, and clinical outcome in severe traumatic brain injury. J Neurosurg 2013;118:1317–28. doi:10.3171/2013.2.JNS121468
15. SAFE Study Investigators. Saline or albumin for fluid resuscitation in patients with traumatic brain injury. N Engl J Med 2007;357:874–84. doi:10.1056/NEJMoa067514
16. Sakellaris G, Kotsiou M, Tamiolaki M, et al. Prevention of complications related to traumatic brain injury in children and adolescents with creatine administration: an open label randomized pilot study. J Trauma 2006;61:322–9. doi:10.1097/01.ta.0000230269.46108.d5
17. Saul TG, Ducker TB, Salcman M, et al. Steroids in severe head injury: A prospective randomized clinical trial. J Neurosurg 1981;54:596–600. doi:10.3171/jns.1981.54.5.0596
18. Skolnick BE, Maas AI, Narayan RK, et al. [A clinical trial of progesterone for severe traumatic brain injury.](http://www.ncbi.nlm.nih.gov/pubmed/25493978) N Engl J Med. 2014 Dec 25;371(26):2467-76. doi: 10.1056/NEJMoa1411090. Epub 2014 Dec 10
19. Temkin NR, Anderson GD, Winn HR, et al. Magnesium sulfate for neuroprotection after traumatic brain injury: a randomised controlled trial. Lancet Neurol 2007;6:29–38.
20. Wright DW, Yeatts SD, Silbergleit R, et al. [Very early administration of progesterone for acute traumatic brain injury.](http://www.ncbi.nlm.nih.gov/pubmed/25493974) N Engl J Med. 2014 Dec 25;371(26):2457-66. doi: 10.1056/NEJMoa1404304. Epub 2014 Dec 10

**References for bacterial meningitis meta-analysis**

1. de Gans J, van de Beek D. Dexamethasone in adults with bacterial meningitis. N Engl J Med 2002;347:1549–56. doi:10.1056/NEJMoa021334
2. Mathur NB, Garg A, Mishra TK. Role of dexamethasone in neonatal meningitis: a randomized controlled trial. Indian J Pediatr 2013;80:102–7. doi:10.1007/s12098-012-0875-9
3. Molyneux EM, Walsh a L, Forsyth H, et al. Dexamethasone treatment in childhood bacterial meningitis in Malawi: a randomised controlled trial. Lancet 2002;360:211–8.http://www.ncbi.nlm.nih.gov/pubmed/12133656
4. Nguyen THM, Tran THC, Thwaites G, et al. Dexamethasone in Vietnamese adolescents and adults with bacterial meningitis. N Engl J Med 2007;357:2431–40. doi:10.1056/NEJMoa070852
5. Odio CM, Faingezicht I, Paris M, et al. The beneficial effects of early dexamethasone administration in infants and children with bacterial meningitis. N Engl J Med 1991;324:1525–31. doi:10.1056/NEJM199105303242201
6. Qazi S a, Khan M a, Mughal N, et al. Dexamethasone and bacterial meningitis in Pakistan. Arch Dis Child 1996;75:482–8.
7. Scarborough M, Gordon SB, Whitty CJM, et al. Corticosteroids for bacterial meningitis in adults in sub-Saharan Africa. N Engl J Med 2007;357:2441–50. doi:10.1056/NEJMoa06571
8. Thwaites GE, Nguyen DB, Nguyen HD, et al. Dexamethasone for the treatment of tuberculous meningitis in adolescents and adults. N Engl J Med 2004;351:1741–51. doi:10.1056/NEJMoa040573
